# The Public Health Leadership and Implementation Academy for Noncommunicable Diseases

**DOI:** 10.5888/pcd16.180517

**Published:** 2019-04-18

**Authors:** Karla I. Galaviz, K.M. Venkat Narayan, Olivia C. Manders, Gabriela Torres-Mejía, Shifalika Goenka, Deborah A. McFarland, K. Srinath Reddy, Rafael Lozano, Laura Magaña Valladares, Dorairaj Prabhakaran, Mohammed K. Ali

**Affiliations:** 1 Rollins School of Public Health, Emory University, Atlanta, Georgia; 2 National Institute of Public Health, Cuernavaca, Morelos, Mexico; 3 Public Health Foundation of India, New Delhi, India; 4 Institute for Health Metrics and Evaluation, Department of Global Health, University of Washington, Seattle, Washington

## Abstract

**Purpose and Objectives:**

Low‐ and middle‐income countries (LMICs) have a large burden of noncommunicable diseases and confront leadership capacity challenges and gaps in implementation of proven interventions. To address these issues, we designed the Public Health Leadership and Implementation Academy (PH-LEADER) for noncommunicable diseases. The objective of this program evaluation was to assess the quality and effectiveness of PH-LEADER.

**Intervention Approach:**

PH-LEADER was directed at midcareer public health professionals, researchers, and government public health workers from LMICs who were involved in prevention and control of noncommunicable diseases. The 1-year program focused on building implementation research and leadership capacity to address noncommunicable diseases and included 3 complementary components: a 2-month online preparation period, a 2-week summer course in the United States, and a 9-month, in-country, mentored project.

**Evaluation Methods:**

Four trainee groups participated from 2013 through 2016. We collected demographic information on all trainees and monitored project and program outputs. Among the 2015 and 2016 trainees, we assessed program satisfaction and pre–post program changes in leadership practices and the perceived competence of trainees for performing implementation research.

**Results:**

Ninety professionals (mean age 38.8 years; 57% male) from 12 countries were trained over 4 years. Of these trainees, 50% were from India and 29% from Mexico. Trainees developed 53 projects and 9 publications. Among 2015 and 2016 trainees who completed evaluation surveys (n = 46 of 55), we saw pre–post training improvements in the frequency with which they acted as role models (Cohen’s *d *= 0.62, *P* <.001), inspired a shared vision (*d *= 0.43, *P* =.005), challenged current processes (*d *= 0.60, *P* <.001), enabled others to act (*d *= 0.51, *P* =.001), and encouraged others by recognizing or celebrating their contributions and accomplishments (*d *= 0.49, *P* =.002). Through short on-site evaluation forms (scale of 1–10), trainees rated summer course sessions as useful (mean, 7.5; SD = 0.2), with very good content (mean, 8.5; SD = 0.6) and delivered by very good professors (mean, 8.6; SD = 0.6), though they highlighted areas for improvement.

**Implications for Public Health:**

The PH-LEADER program is a promising strategy to build implementation research and leadership capacity to address noncommunicable diseases in LMICs.

**Summary**

**What is already known about this topic?**

Low- and middle-income countries (LMICs) face limited implementation research and leadership capacity to translate evidence into programs and policies addressing noncommunicable diseases.

**What is added by this report?**

We developed a 1-year training program to build implementation research and leadership capacity among public health professionals in LMICs. From 2013 through 2016, 90 professionals from 12 countries were trained, and results from an evaluation of the program are promising.

**What are the implications for public health practice?**

This training program can contribute to strengthen the global-health workforce and its ability to lead and implement proven strategies to address noncommunicable diseases in LMICs.

## Introduction

Cardiovascular disease, diabetes, cancer, mental illness, and chronic respiratory diseases threaten the health and economic well-being of individuals and populations alike (1). These noncommunicable diseases account for 71% of all deaths worldwide and are a serious public health threat in low- and middle-income countries (LMICs), which is where 78% of deaths from noncommunicable diseases occur (2). Noncommunicable diseases are also a barrier to economic development in LMICs (3) because they occur most frequently in these countries’ most active age group (25–64 y), resulting in loss of human capital and productivity.

LMICs confront major challenges to address the growing burden of noncommunicable diseases. Aside from limited health financing, scarcity of local health research evidence, and heterogeneity in access to health care and care delivery, LMICs face 2 additional and often underrecognized challenges: limited implementation research and limited leadership capacity among the public health workforce to translate research evidence into policy and practice. For instance, limited capacity hinders implementation of proven strategies to prevent noncommunicable diseases (eg, lifestyle modification, tobacco control, hypertension control) (4–6) and manage them (eg, diet modification, quality diabetes care, access to low-cost medication) (7,8). Furthermore, implementation of proven strategies requires strong leaders to address challenges (eg, political issues) and opportunities associated with disseminating evidence (9) and to make large-scale change (10). Lack of leadership can be a barrier to evidence-based public health practice and can affect public health system performance (9,11). Few, if any, LMICs have sufficient capacity for implementation research to enhance systems and integrate the preventive and curative services required to address noncommunicable diseases.

Though training can improve implementation research and leadership skills in the public health workforce, existing training programs have targeted these skill sets separately and mainly among professionals from high-income countries (12–16). Other training programs have included professionals from several countries, but these focused solely on improving implementation research capacity (17,18). Because there are no training opportunities for LMICs that target both implementation research and leadership capacity, we designed the Public Health Leadership and Implementation Academy (PH-LEADER) for noncommunicable diseases. The program aimed to build the leadership skills and capacity for implementation research of 21st century global leaders in prevention and control of noncommunicable diseases. The program was developed and implemented through a collaboration between Emory University in the United States, the National Institute of Public Health of Mexico, and the Public Health Foundation of India. The 1-year training program was implemented from 2013 through 2016 and 4 cohorts of trainees participated. We previously reported the baseline characteristics of the 2013–2015 trainees (19); in this article, we report results from a program evaluation among the 4 consecutive cohorts of trainees (2013–2016).

## Purpose and Objectives

The objective of this program evaluation was to assess the quality and impact of the PH-LEADER training program. We define training as a set of didactic, theoretical, and instructional methods focused on enhancing implementation research and leadership skills. PH-LEADER was developed collaboratively by public health experts from Emory University, the National Institute of Public Health in Mexico, and the Public Health Foundation of India with the goal of building leadership and implementation research capacity to address noncommunicable diseases in 12 countries (Table 1). These countries were selected because they are experiencing a growing burden of noncommunicable diseases (20) and confront challenges of capacity, dearth of local evidence, gaps in implementation of proven interventions (4–8), and heterogeneity in health care access and delivery (20,21). Though India and Mexico were the primary focus of this training program, participants from other LMICs were also trained. PH-LEADER was funded by the National Institutes of Health’s Fogarty International Center.

**Table 1:** Characteristics of Trainees Enrolled In The 1-Year Program By Cohort Year, Public Health Leadership and Implementation Academy, 2013–2016^a^

Characteristic	2013 Cohort (n = 14)	2014 Cohort (n = 21)	2015 Cohort (n = 31)	2016 Cohort (n = 24)	All (n = 90)
**Age, mean (standard deviation), y**	39.6 (6.6)	38.1 (6.6)	38.4 (7.7)	38.9 (7.3)	38.8 (7.1)
**Male**	6	10	20	15	51
**Position**					
Researcher	5	7	11	5	28
Professor	5	8	7	6	33
Leadership	2	4	7	13	26
Consultant	2	2	6	1	11
**Institution**					
Academic	4	9	6	7	26
Government	4	6	9	4	23
Foundation	1	5	5	9	20
Civil society	0	0	2	0	2
Health care	3	0	9	4	16
**Country**					
India	6	10	13	16	45
Mexico	6	8	6	6	26
Brazil	1	1	—b	—b	2
Colombia	1	1	—	—b	2
Kingdom of Saudi Arabia	—b	—b	6	—b	6
Barbados	—b	—b	2	—b	2
Georgia	—b	—b	1	—b	1
Guatemala	—b	—b	1	—b	1
Malawi	—b	1	—b	—b	1
Liberia	—b	—b	1	—b	1
Burkina Faso	—b	—b	—b	1	1
United States	—b	—b	1	—b	1

a Values indicate number of participants in that group or country unless otherwise indicated.

b Program had no participants from this country for that year.

PH-LEADER was directed at midcareer, high-potential health researchers, public health professionals, and government public health workers affiliated with the National Institute of Public Health in Mexico, the Public Health Foundation of India, and ministries of health or strategic partner institutions in other LMICs. The training program was advertised internally at the National Institute of Public Health in Mexico and the Public Health Foundation of India and externally via email listservs, websites, and word of mouth. To promote equal opportunity, we focused participant recruitment strategies on women, low socioeconomic populations, geographically underserved areas, and professionals with limited access to similar training opportunities. We also asked in-country partners to find and encourage applications from women and to select equal numbers of women and men where possible. This was done with careful consideration to equity and balance in subject expertise where possible; this also helped select candidates from institutions that may otherwise have been overlooked for this training opportunity.

Individuals interested in participating submitted an application in English that included a letter of intent, their curriculum vitae, and 2 letters of support from their home institution. The application was screened independently by 2 members of a review committee and ranked by using a simple scoring system from 1 to 10, with 1 being the lowest score. The scoring system was designed to identify applicants with high potential based on years of experience, likelihood of moving into a position of influence in their organization, and potential to affect public health in their home country. We selected participants with the highest scores while also considering diversity in sex, institution, and area of expertise.

## Intervention Approach

PH-LEADER program theory and content are described in detail elsewhere (19). Briefly, PH-LEADER was a 1-year training program aimed at fostering implementation research and leadership capacity among mid-career health professionals, researchers, and government public health workers from LMICs. The program was based on the evidence-based public health framework (9) and focused on building capacity to make decisions on the basis of the best available evidence, using data and information systems systematically, applying program-planning frameworks, engaging the community in decision making, conducting sound evaluation, and disseminating findings. The program also focused on fostering public health leadership competencies (22) because effective leadership is required to make decisions, shape organizational culture, and apply scientific knowledge to public health problems (9,11,23).

The 1-year program comprised 3 complementary parts. The first was a 2-month in-country preparation period that consisted of webinars and reading materials aimed at laying the knowledge and work-dynamic foundation needed for the course. This was followed by a 2-week, in-person, executive-style course delivered at Emory University in Atlanta. An immersive summer course format was used because it maximized opportunities for trainees and faculty to interact, provided practical learning opportunities, and promoted short-term improvements in trainee skills (16,24). The summer course was organized in 3 learning modules: one focused on analytical skills, a second focused on implementation research skills, and a third focused on public health leadership competencies (9,22). As part of the leadership module, trainees completed the Birkman Method assessment (25) to help them develop self-awareness, facilitate interpersonal engagement among trainees, and identify and resolve intrapersonal and interpersonal conflicts. The summer course was delivered by a faculty with members from India, Mexico, the United States, and the United Kingdom with broad expertise in program development and implementation, public health research, organizational management, and public health policy. (Training materials are available from the corresponding author.)

The summer course was followed by a structured 9-month, in-country mentored phase that involved distance learning through monthly webinars and participant-led implementation projects (Box). Interactive webinars were used to reinforce learning and promote trainee engagement during the in-country program phase (26,27). Mentorship is critical to career development among junior investigators (28) and to implementation research training (14); thus, it was offered to all trainees to promote in-country project completion and trainee career development. Mentors were largely public health academics from our partner institutions in Mexico and India; trainee–mentor interactions were ad hoc and dependent on initiative from the trainee.

**Box. Public Health Leadership and Implementation Academy**

**Course Preparation (in country, 2 months)**

Pre-course orientation webinars

Preparatory readings and materials

**In-Person Course (Atlanta, 2 weeks)**

**Module 1 — Analytical Methods**

Basics of epidemiology

Basic economics

Surveillance

Economic evaluation

Data integration and information systems

Systematic reviews

Research ethics

Health systems research

**Module 2 — Implementation Research**

Implementation science frameworks

Quasi-experiments and natural experiments

Organizational design

Health financing

Qualitative research methods

Structure of health care systems

Health policy

Intervention design, implementation, and evaluation

**Module 3 — Leadership**

Emotional intelligence

Networking and team building

Quality improvement

Organizational culture and management

Managing people

Mentoring and coaching

Managing change

Vision

**Mentored Period (in-country, 9 months)**

Monthly webinars (distance learning)

Meet and learn from the mentor

Develop and implement the project (with mentor)

Alumni network

The program was expected to have an effect in the short term by improving trainee implementation research skills to develop, implement, and test strategies addressing noncommunicable diseases and by equipping them with leadership skills to lead and manage teams to implement, disseminate, and scale such strategies. In the long term, the program is expected to have a ripple effect through trainees influencing organizational culture and program effectiveness and through their mentoring of young public health professionals in their countries (Figure). This article presents the results from the evaluation of the program’s short-term effect.

**Figure:**
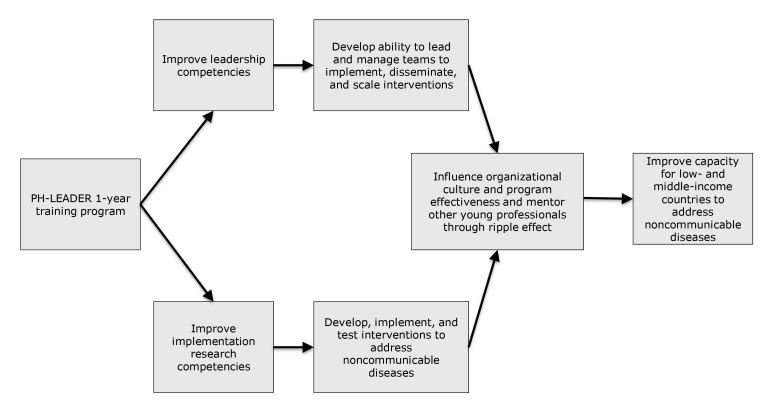
Public Health Leadership and Implementation Academy program model.

## Evaluation Methods

The program evaluation protocol was reviewed and deemed exempt by the Emory University Institutional Review Board. Four 1-year cohorts were accepted into the program, and courses were delivered annually from 2013 through 2016. Among all trainees, we collected demographic information at the time of enrollment and monitored project and program outputs, such as project and program development, conference presentations, publications, and additional funding obtained that was associated with program participation.

We conducted a pretest–posttest single-group evaluation among 2015 and 2016 trainees to assess program effects on leadership practices and perceived competence to conduct implementation research. Leadership practices were assessed by using the Student Leadership Practices Inventory (29), which includes 30 statements assessing the frequency with which trainees engage in leadership practices, with answers anchored in a 5-point Likert scale ranging from 1 (rarely) to 5 (frequently). Perceived competence in conducting implementation research in the trainee’s home organization was assessed by using a scale based on established methodologies (30) that included 5 items with answers anchored in a 7-point scale ranging from 1 (strongly agree) to 7 (strongly disagree). An online survey containing these measures was sent to trainees before training and at training completion by using the Research Electronic Data Capture (REDCap) system (31).

We also assessed satisfaction among 2015 and 2016 trainees with the summer course they attended at Emory University. Trainees completed this assessment through short online evaluation forms for each training session they attended. In these forms, trainees rated the usefulness of information presented from 0 (very useless) to 10 (very useful), and the overall caliber of the content and faculty in each session from 0 (bad) to 10 (excellent).

For analyses, data were summarized as means and standard deviations or frequencies and percentages. Paired sample *t* tests were used to assess changes in leadership practices and perceived competence from baseline to training completion. Cohen’s *d* was used to estimate effect sizes for leadership practices and perceived competence changes (*d* = mean 1 −mean 2 ÷ pooled standard deviation). Values greater than 0.5 were deemed as meaningful effect sizes, indicating pre–post means in leadership practices, and perceived competence scores differ by half a standard deviation. We used the Bonferoni correction method to adjust for multiple comparisons; hence, the significance level was set at α < .006. Data analyses were conducted by using the Statistical Package for Social Sciences, version 23 (IBM Corp).

## Results

Over the 4 years of program delivery, 270 health care and public health professionals, researchers, and government public health workers working in noncommunicable disease submitted applications, and 95 were accepted in the program. Of these, 90 attended and completed the program (mean age 38.8; 57% male); 45 (50%) were from India, 26 (29%) were from Mexico, and the remainder were from other countries (Table 1). Twenty-six (29%) trainees were affiliated with academic institutions and 23 (26%) with government institutions.

The 1-year program cost on average $4,000 USD per participant; this cost was estimated for India and Mexico participants only. Cost included travel from India or Mexico (~$1,200), hotel cost for 2 to 3 weeks (~$1,800), the Birkman Method assessment ($150 per trainee), food and snacks over the course ($150 per trainee), course materials (eg, books, swag, bag [$200 per trainee]), and heavily discounted honoraria for some faculty members (~$500, though most faculty time was provided in kind). Accepted participants from India and Mexico were supported by program funds, which were sufficient to fund 71 trainees over the 4 years of program delivery. Participants from other countries (n = 19) either obtained support in their country to cover program cost or paid a tiered fee based on country-income group of the participant.

As part of the training program, participants across the 4 cohorts (2013–2016, n = 90) developed 53 in-country projects; 38 were completed, and 15 are ongoing. These included the development of evidence-based health interventions (n = 8), strategies to improve local health care systems (n = 4), approaches to inform policy or assess its effect (n = 7), strategies to inform or change clinical practice (n = 14), strategies to improve surveillance or measurement of health outcomes (n = 8), and epidemiological studies (n = 12). Of these, 18 trainees obtained funding from in-country or international agencies to complete their projects. In terms of academic products derived from these projects, 21 abstracts were presented at scientific conferences; as of November 2018, 13 articles were in preparation, 9 articles were under review in scientific journals, and 8 were published (Table 2). Also, 1 trainee published a book chapter.

**Table 2:** Project and Program Output by Cohort Year, Public Health Leadership and Implementation Academy, 2013–2016^a^

Output^b^	2013 Cohort (n = 14)	2014 Cohort (n = 21)	2015 Cohort (n = 31)	2016 Cohort (n = 24)	All (n = 90)
**Projects**					
Developed	10	15	13	15	53
Ongoing	1	1	3	10	15
Completed	9	14	10	5	38
**Conference presentations **	6	5	7	3	21
**Publications**					
In preparation	2	3	5	3	13
Under review	3	2	2		9
Published^b^	3	4	1	1	9
Funding/support obtained	7	2	4	5	18

a Values indicate number of participants in that category/group.

b Includes a book chapter published by 1 trainee.

Of the 55 participants attending the 2015 and 2016 training programs, 46 completed the pre–post program evaluations. Mean participant age was 38.5 years (SD, 8.2) and 28 (60%) were male. We found significant improvements in the frequency with which trainees reported engaging in leadership practices from baseline to end of the training with moderate effect sizes (Table 3). Trainees reported an increase in the frequency with which they acted as role models (Cohen’s *d* = 0.62, *P* <.001), inspired a shared vision (*d* = 0.43, *P* =.005), challenged current processes (*d* = 0.60, *P* <.001), enabled others to act (*d* = 0.51, *P* =.001), and encouraged the heart (encouraged others by recognizing and celebrating their contributions and accomplishments [*d* = 0.49, *P* =.002]). We observed no significant improvements in perceived competence to conduct implementation research in trainees’ home institutions.

**Table 3:** Changes in Leadership Practices and Perceived Competence Among Trainees (N = 46) From Baseline to Training Completion in 2015 and 2016 Cohorts, Public Health Leadership and Implementation Academy, 2013–2016^a^

Variable	Baseline (n = 46)	Post-training (n = 46)	*d* b	*P *Value^c^
**Leadership practices^d^**				
Model the way	3.7 (0.6)	4.0 (0.6)^d^	0.62	<.001
Inspire a shared vision	3.7 (0.6)	4.0 (0.7)^d^	0.43	.005
Challenge the process	3.8 (0.7)	4.1 (0.5)^d^	0.60	<.001
Enable others to act	4.1 (0.5)	4.4 (0.4)^d^	0.51	.001
Encourage the heart	3.9 (0.6)	4.2 (0.6)^d^	0.49	.002
**Perceived competence^e^**				
Feel conducting implementation research is easy	4.7 (1.2)	4.5 (1.4)	—f	.34
Is confident in conducting implementation research	2.7 (1.5)	2.9 (1.8)	—f	.52
Perceive conducting implementation research is outside their control	3.6 (1.9)	3.8 (2.0)	—f	.59
Perceive conducting implementation research is within their control	4.1 (1.8)	4.1 (1.8)	—f	.94

a Only participants who completed the 2015 and 2016 training programs completed this evaluation. Values are mean (standard deviation).

b *d* = Cohen’s effect size measure for paired sample *t* test. This is computed by obtaining the difference between the 2 group means and dividing it by the average of their standard deviations. A d of 1 indicates means differ by 1 standard deviation, where 0.2 is considered a small effect size, 0.5 a medium effect size, and 0.8 a large effect size.

c Significantly different from baseline, *P* <.01 calculated by using Bonferoni adjustment and paired sample *t* tests.

d Measured on a 5-point scale ranging from 1, rarely, to 5, frequently.

e Measured on a 7-point scale ranging from 1, strongly agree, to 7, strongly disagree.

f Effect size measure not applicable because of lack of pre–post changes.

Overall, 33 to 41 training sessions lasting between 90 and 120 minutes each were delivered each year over 2 weeks. Trainees regarded course sessions as useful (mean, 7.5; SD, 0.2), with a very good content (mean, 8.5; SD, 0.6) and delivered by very good professors (mean, 8.6; SD, 0.6). When training modules were assessed separately, the leadership module achieved the highest ratings (Table 4). Although trainees generally reported a high level of satisfaction with the curriculum immediately following the summer course, they also highlighted areas for improvement. For instance, trainees suggested that more information about the structure of the course be provided up front, that the sequencing of the curriculum be revised (eg, methods, then leadership, then implementation) and that the workload and duration of the summer course be reduced.

**Table 4:** Ratings for Each Training Module Delivered During the 2015 and 2016 Summer Courses, Public Health Leadership and Implementation Academy, 2013–2016^a^

Module	Ratings Across Sessions^a^		
	Usefulness^b^	Content^c^	Professor^c^
Analytical methods	7.4 (0.2)	8.2 (0.5)	8.4 (0.6)
Implementation research	7.4 (0.2)	8.3 (0.5)	8.4 (0.6)
Leadership	7.7 (0.1)	9.0 (0.3)	9.1 (0.3)

a Values are mean (standard deviation); 41 sessions were delivered in 2015 and 33 in 2016. Ratings are the average of all sessions delivered in the 2 years. Only participants who completed the 2015 and 2016 summer courses completed this evaluation.

b Measured on a 10-point scale ranging from 0, very useless, to 10, very useful.

c Measured on a 10-point scale ranging from 0, bad, to 10, excellent.

## Implications for Public Health

Leadership and implementation science are some of the most overlooked yet most essential paradigms in public health (9,32). The PH-LEADER program focused on building leadership and implementation research capacity among 21st century public health professionals from LMICs. Over 4 years, 90 public health professionals, researchers, and government public health workers from 12 LMICs were trained; the program evaluation showed training improved leadership practices though not perceived competence for implementation research. The training also promoted the development of research and programmatic projects addressing noncommunicable diseases in LMICs and was well accepted by trainees.

Our findings align with those from evaluations of similar programs. For instance, the National Public Health Leadership Institute program was found to improve collaborative leadership and to promote the development of knowledge-sharing and problem-solving networks (12). Similarly, the Leadership and Organizational Change for Implementation program was found to improve leadership for evidence-based practice implementation (13). Regarding implementation research, the Evidence-Based Public Health program was found to improve knowledge, skill, and ability to implement evidence-based public health among public health academics and practitioners in the United States and abroad (18). Also, the US Training for Dissemination and Implementation Research was found to promote the development of new implementation research grant proposals (16), and the Mentored Training for Dissemination and Implementation Research in Cancer was effective in improving several implementation research competencies (14).

We found no improvements in perceived competence for conducting implementation research, which may be due to a measurement issue. The “perceived competence” measure we used is more akin to control beliefs (ie, about behavior barriers/facilitators) and intentions (ie, motivation) than to actual competence (ie, skills, knowledge, experience). Indeed, a study among medical students found perceived competence in evidence-based medicine did not correlate well with objectively assessed competence (33). A more objective measure of competence in our program would be assessing changes in the degree to which trainees conducted implementation research from baseline to training completion. Although we only assessed this at training completion, results are encouraging: as of November 2018, 38 in-country projects were completed, 15 were ongoing, and 8 manuscripts and 1 book chapter were published (34–42). These achievements are evidence of the program’s potential to energize trainees’ careers, contribute to their professional development, and nurture competence to address noncommunicable diseases.

The 4-year implementation of PH-LEADER taught us several lessons. Through implementation and participant feedback, we learned that the program needed constant refinement, and it did evolve over time. Part of that evolution are current efforts to design a shorter version of the program to be delivered in trainees’ home countries. As previously noted (43), another challenge is to keep up with the rapidly advancing and still evolving field; thus, curriculum content and training methods need continuous revision and adaptation. Regarding program evaluation, we learned that some of the traditional metrics used in academia (eg, number of publications) do not adequately characterize the effect of a leadership program, especially in global health contexts (44). Many leadership endeavors (eg, managing teams) are not accounted for in traditional academic metrics; as such, innovative ways to evaluate program effects on different areas (eg, academia, career development, policy) and using novel methods to assess these (eg, video call interviews) are needed.

We also learned that empathy, respect, and acceptance were essential for promoting positive communication and interactions. The 3-way collaboration among Emory University, Mexico’s National Institute of Public Health, and the Public Health Foundation of India enabled exchange of ideas, broadening of disciplines, and exposure to different research cultures and systems. The program offered an open forum for expression of ideas and fostered cross-cultural exchange and discussion of public health issues. As in previous implementation research training programs (43), we leveraged participant diversity by building strong international, collaborative networks and peer-to-peer mentorship across countries. We also learned that bringing together middle-level decision-makers, researchers, and practitioners from across countries and institutions was a fruitful strategy that facilitated cross-learning and implementation training in terms of sharing experience of best practices. This also expanded trainee networks and led to the development of several promising collaborative projects in LMICs.

Finally, we learned that strategies to promote the sustainability of the program are needed. Obtaining funding to sustain training programs is a major challenge identified in dissemination and implementation research training programs (43). One potential strategy is seeking funding from local governments, private philanthropies, or foundations interested in investing in the local public health work force and in addressing noncommunicable diseases. Shortening the program duration and delivering it in-country by using existing resources is another potential sustainability strategy we have pilot-tested in Mexico with promising results. The use of technology for distance learning, which was instrumental to deliver the in-country portion of our program, is another strategy worth exploring to sustain the program. Evidence from the United States shows distance learning is an effective, wide-reach strategy to build evidence-based decision making capacity (27); thus, using distance learning strategies could help increase the scale and reach of the PH-LEADER program.

The present program evaluation should be interpreted in light of several limitations. The program design prevented us from comparing the training course with other approaches or with no training at all. Leadership practices and perceived competence for implementation research were measured by using self-reports, which are prone to social desirability bias and may bias estimates of training effects toward larger effects. Finally, the participant sample was self-selected; thus, these findings are only applicable to those who participated in the program and are generalizable only to cohorts of motivated public health professionals and researchers linked to these opportunities.

The PH-LEADER program is a promising strategy to build leadership and implementation research capacity among 21st century public health professionals from LMICs. To our knowledge, our program is the first to addresses both skill sets jointly, and results from the initial program evaluation are promising. Findings from this evaluation are being used to inform adaptations of the program and to pilot its delivery in LMIC settings using existing resources. Real-world implementation of effective strategies to address noncommunicable diseases can be pursued through training effective public health leaders. If adapted for wide-scale and low-cost delivery, PH-LEADER can contribute to strengthen the global-health workforce and its ability to lead and implement proven strategies to address noncommunicable diseases in LMICs.
